# Definition of miRNAs Expression Profile in Glioblastoma Samples: The Relevance of Non-Neoplastic Brain Reference

**DOI:** 10.1371/journal.pone.0055314

**Published:** 2013-01-29

**Authors:** Michela Visani, Dario de Biase, Gianluca Marucci, Cristian Taccioli, Agostino Baruzzi, Annalisa Pession, the PERNO Study group

**Affiliations:** 1 Department of Experimental Pathology, University of Bologna, Bologna, Italy; 2 Department of Hematologic and Oncological Sciences, University of Bologna, Bologna, Italy; 3 Department of Cancer Biology, Paul O'Gorman Cancer Institute, University College London, London, United Kingdom; 4 Department of Biomedical & Neuromotor Sciences, University of Bologna and IRCCS Istituto delle Scienze Neurologiche di Bologna, Bologna, Italy; Instituto de Investigación Sanitaria INCLIVA, Spain

## Abstract

Glioblastoma is the most aggressive brain tumor that may occur in adults. Regardless of the huge improvements in surgery and molecular therapy, the outcome of neoplasia remains poor. MicroRNAs are small molecules involved in several cellular processes, and their expression is altered in the vast majority of tumors. Several studies reported the expression of different miRNAs in glioblastoma, but one of the most critical point in understanding glioblastoma miRNAs profile is the comparison of these studies. In this paper, we focused our attention on the non-neoplastic references used for determining miRNAs expression. The aim of this study was to investigate if using three different non-neoplastic brain references (normal adjacent the tumor, commercial total RNA, and epileptic specimens) could provide discrepant results. The analysis of 19 miRNAs was performed using Real-Time PCR, starting from the set of samples described above and the expression values compared. Moreover, the three different normal RNAs were used to determine the miRNAs profile in 30 glioblastomas. The data showed that different non-neoplastic controls could lead to different results and emphasize the importance of comparing miRNAs profiles obtained using the same experimental condition.

## Introduction

MicroRNAs (miRNAs) are small RNA molecules involved in several cellular processes. Briefly, these small RNAs regulate proteins expression by binding target mRNAs with a perfect or imperfect complementarity [1]. The miRNAs expression analysis could be performed using different techniques, such as microarray assays or Real-Time PCR. Regardless of the chosen approach, one of the most important decisions before analyzing miRNAs profile (as well as for mRNAs expression studies) is the selection of a reference control. The availability of non-neoplastic specimens used as reference is often subordinated to understudied tissue. Differently from what happens for other tissues such as breast or lung [2]–[4], obtaining brain specimens from healthy subjects is very difficult and, therefore, finding a suitable non-neoplastic control for the analysis of RNA in brain neoplasia still remains a big issue. Moreover, for surgical neoplastic brain samples, the non-neoplastic area is usually absent, very limited, or adjacent the tumor, as for glioblastoma (GBM).

This study was conducted within the PERNO (Progetto Emiliano-Romagnolo di Neuro-Oncologia) project. One of the goals of PERNO is to investigate the role of miRNAs in GBM. In a previous paper [5], we demonstrated the feasibility of miRNAs analysis in brain specimens starting from formalin-fixed and paraffin-embedded tissues (FFPE), as well as in fresh/frozen samples. In literature, there are at least three different specimens used as normal reference for miRNAs analysis in brain samples: the normal area adjacent the tumor [6]–[8], one of the available commercial references (FirstChoice® Human Brain Reference RNA, Ambion) [9], [10], and the tissue removed in epileptic patients [11], [12]. Before looking for miRNAs profile in GBM, we decided to deeply investigate the miRNAs expression values in these three different non-neoplastic RNAs.

The aim of this study was to compare three different references used as non-neoplastic control for miRNAs analysis in GBM (the normal area adjacent the tumor, a commercial reference, and the tissue removed in epileptic patients) by investigating the expression levels of nineteen miRNAs. In order to clarify if the choice of non-neoplastic samples could influence the miRNAs analysis in GBM, the miRNAs profiles of thirty GBMs were also investigated using each one of the three references as control.

## Materials and Methods

### Ethic Statement

The study was approved by Ethic Committee of Azienda Sanitaria Locale di Bologna (number of study 08075, protocol number 139/CE of 5^th^ February 2009, Bologna, Italy). All patients signed a written consent for molecular analysis and anonymous data publication for scientific studies, and all information regarding the human material used in this study was managed using anonymous numerical codes.

### Selection of Cases

MicroRNAs expression analysis was performed using a commercial brain reference (FirstChoice® Human Brain Reference RNA, Ambion, Austin, TX, USA), 15 cases of normal samples adjacent the tumor and 15 cases of polar temporal cortical (PTC) specimens removed in patients submitted to surgery (tailored polar anterior temporal resection along with uncus-amygdalohippocampectomy) for drug-resistant epilepsy. All the cases were retrieved at Bellaria Hospital (Section of Pathology, Bologna, Italy), and normal samples adjacent the tumor specimens were included within the PERNO project.

#### Normal adjacent the tumor

Normal adjacent the tumor tissues were retrieved at a distance between 1 and 2 cm from the margin of 15 primary FFPE GBMs. Patients were 8 males and 7 females, aged 50 to 75 years (mean 62.7 yrs). All samples were diagnosed as GBM according to the 2007 WHO criteria [13]. Thirty samples were also used for the GBMs profile (see below).

#### Commercial reference

The FirstChoice® Human Brain Reference RNA from Ambion was used. According to the manufacturers' data sheet, it was obtained from several normal brain regions (meaning free of brain pathology) of 23 donors, 13 males and 10 females, aged 23 to 86 (mean 69.7 yrs). FirstChoice® is certified to contain small RNAs, including miRNAs.

#### Epileptic tissue

Fifteen FFPE PTC samples were randomly selected. Epileptic patients were 7 males and 8 females, aged 25 to 52 years (mean 39.7 yrs). All of them presented drug-resistant anteromedial temporal lobe epilepsy. Histologically, eleven cases showed focal cortical dysplasia while four patients had hippocampal sclerosis. None of them were affected by a neoplastic lesion, including GBM. The tissue used for miRNAs extraction was taken from the temporal lobe cortex.

#### Glioblastoma

Thirty patients were selected for determining GBMs profile using the three different non-neoplastic references. All specimens were primary GBMs, and patients had not undergone neoadjuvant therapy before surgery. Patients were 14 males and 16 females, aged 42 to 75 years (mean 63.3 yrs). All samples were diagnosed as GBM according to the 2007 WHO criteria [13].

### miRNAs Analysis

The hematoxylin and eosin (H&E) sections were reviewed by a pathologist (GM) to select the more informative block. Four 20 μm-thick sections were cut, followed by one H&E control slide. The area selected for the analysis was marked on the control slide to ensure, whenever possible, greater than 90% content of glial cells (normal adjacent the tumor and epileptic specimens) or neoplastic cells (glioblastoma samples).

Nineteen miRNAs (miR-7, miR-9, miR-9*, miR10a, miR10b, miR-17, miR-20a, miR-21, miR-26a, miR-27a, miR-31, miR-34a, miR-101, miR-137, miR-182, miR-221, miR-222, miR-330, miR-519d) were studied according to their role in GBM and because of their previous technical validation in order to determine the feasibility of analysis starting from FFPE tissues [5]. Three small RNAs (RNU49, U54, miR-103) were used as internal control [5]. The miRNAs extraction and analysis were performed as previously described [5]. Briefly, RNA was retro-transcribed using the NCode miRNA First-Strand cDNA Synthesis and qRT-PCR Kits (Invitrogen, Carlsbad, CA, USA), and miRNAs expression was evaluated using an AB7000 machine (Applied Biosystem, Foster City, CA, USA). Each miRNA was run twice per each sample. Considering that commercial reference was a pool of RNA obtained from normal brain, it was analyzed three times (technical replicates).

### Statistical Analysis

Expression values and fold changes were obtained by relative quantification and 2^−ΔΔCT^ method [14] using the DataAssist 2.0 Tool (Applied Biosystem, Foster City, CA, USA). In order to determine miRNAs profile obtained in GBM, the median fold-change of each miRNA in the 30 GBM samples was compared with “control samples” (15 epileptic specimens, 14 normal adjacent tissues and 1 commercial reference). A GBM/Control ratio <−2.0 means that miRNA was downregulated, while a ratio ≥2.0 means that miRNA was upregulated. Statistical analysis of miRNAs expression was performed using GraphPad Prism 5.0 tool. Gaussian distribution was evaluated by Shapiro-Wilk Test. Correlation analysis between miRNAs expression in the three different groups were performed using Spearman correlation test. For comparing the expression levels of each miRNA obtained in the three groups, Kruskal-Wallis and Mann-Whitney tests were used. Level of significance was p<0.05 for all the statistical analysis.

## Results

All the samples, except one normal adjacent the tumor specimen, gave sufficient quantity of miRNAs for performing the analysis.

### miRNAs Analysis in Normal References

Distribution for normal adjacent the tumor, commercial reference, and epileptic groups was not Gaussian as demonstrated by the Shapiro Test (p<0.001). For this reason, we used non-parametric statistical tests.

All Spearman correlation values between the expression levels of each miRNAs obtained in the three groups were above 0.65 (p<0.0001) (Table 1).

**Table 1:** Spearman correlation values between three groups (p<0.0001).

	*Normal Adjacent Tumor*	*Ambion Brain Reference*	*Epileptic Tissue*
***Normal Adjacent Tumor***	/	0.724	0.702
***Ambion Brain Reference***	0.724	/	0.848
***Epileptic Tissue***	0.702	0.848	/

**Figure 1:**
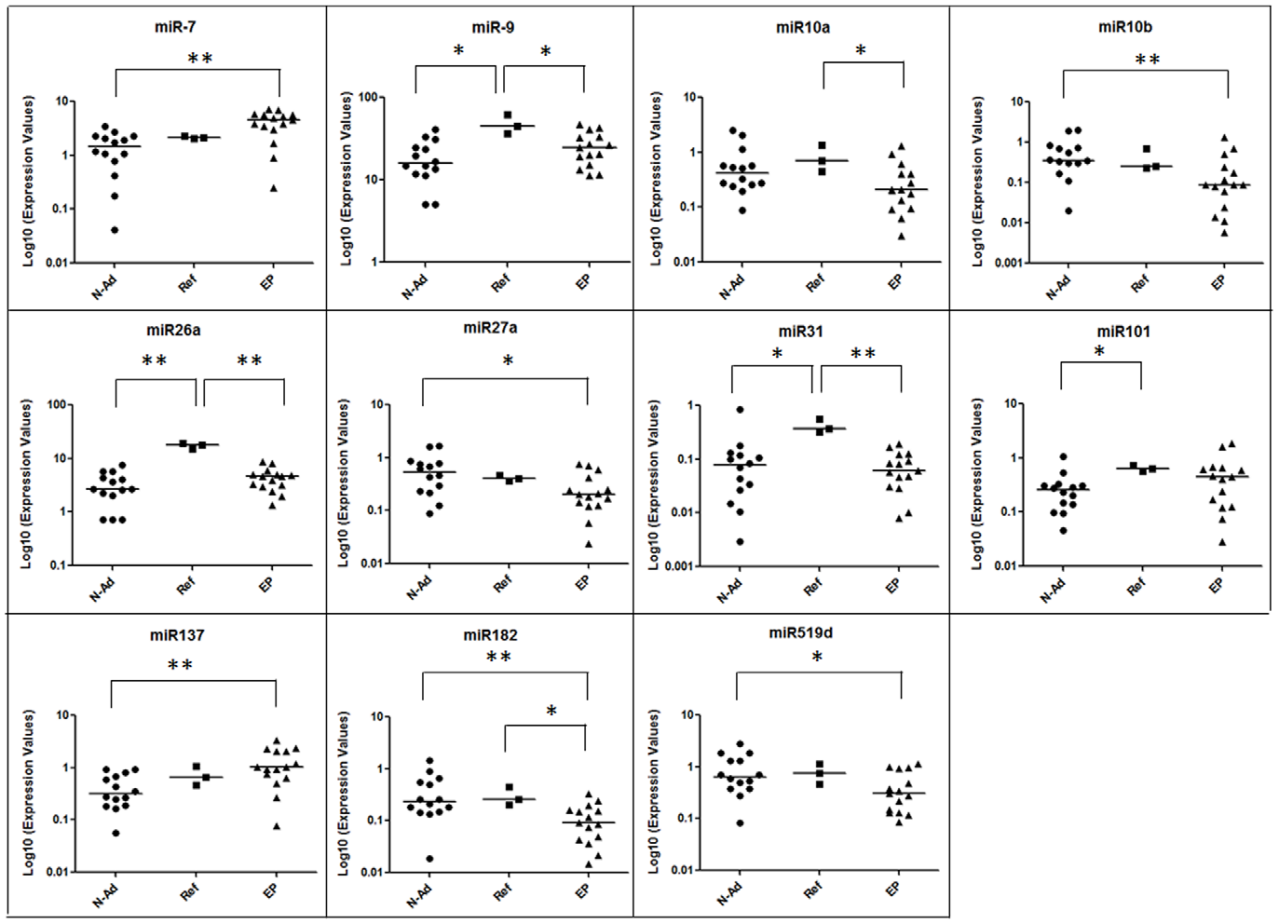
Differences in miRNAs expression. Scatter plots show miRNAs significantly different between groups. Bars indicate median values. * p<0.05, ** p<0.01 according to Mann-Whitney test. The representation of commercial reference (a pool of normal brain RNA) indicates technical variability, while scatter plots of Normal adjacent the tumor and epileptic specimens show individual variability. N-Ad, Normal adjacent the tumor; Ref, Commercial reference; EP, epileptic.

While comparing the median expression values obtained in the three different groups, we observed statistical significant differences (p<0.05) in 9 miRNAs: miR-7, miR-9, miR-10a, miR-10b, miR-26a, miR-27a, miR-31, miR-137, and miR-182. For the others, no significant differences were observed (Table S1). Moreover, the Mann-Whitney test, performed considering groups in pairs, revealed statistical significant differences even in miR-101 and miR-519d, as shown in Figure 1. It should be considered that the variability observed in normal adjacent the tumor and in epileptic specimens is a biological variability, while the one observed in commercial reference (a pool of normal brain RNA) is a technical variability.

### GBM Profile

The differences observed when comparing expression values of miRNAs in the three different references led us to further investigate if the choice of non-neoplastic control could give discrepant results in analyzing GBM miRNAs profile. For this reason, we compared the profile of the 19 miRNAs in thirty GBMs matched with the three different non-neoplastic brain references (normal adjacent the tumor, commercial reference, and epileptic tissue).

Using different non-neoplastic reference groups resulted in different GBM miRNAs expression profiles (Table 2 and Figure S1). For example, miR-17 was up-regulated (FC ≥2.0) in GBM when matched with Ambion reference, but was not deregulated when matched with normal adjacent the tumor or epileptic tissue; miR-31 was down-regulated (FC <−2.0) in GBMs matched with Ambion reference and normal adjacent the tumor, but not deregulated when matched with epileptic tissue. Other miRNAs with different expression status were miR-10a, miR-10b, miR-20a, miR-26a, miR-27a, miR-34a, miR-101, miR-182, miR-221, miR-222, and miR-330, as shown in Table 2.

**Table 2:** MiRNAs profile in 30 GBMs compared with the 3 different non-neoplastic references.

miRNAs	Normal adjacent Tumor			Ambion Reference			Epileptic		
	*Median FC ± Median Error*	*Status* a	*N. of cases^b^*	*Median FC ± Median Error*	*Status* a	*N. of cases^b^*	*Median FC ± Median Error*	*Status* a	*N. of cases^b^*
**miR-7**	−7.377±0.257	DOWN	24/30	−11.086±0.117	DOWN	27/30	−23.753±0.055	DOWN	28/30
**miR-9**	1.679±0.671	=	14/30	−1.403±0.194	=	18/30	−1.331±0.290	=	20/30
miR-9*	1.855±1.509	=	14/30	1.684±0.936	=	18/30	1.521±0.884	=	18/30
**miR-10a**	1.296±0.494	=	16/30	1.074±0.280	=	18/30	2.436±0.954	UP	19/30
**miR-10b**	1.844±1.111	=	9/30	3.105±1.157	UP	21/30	4.688±2.699	UP	23/30
**miR-17**	1.660±1.620	=	18/30	2.038±1.358	UP	16/30	1.960±1.360	=	14/30
**miR-20a**	2.396±0.729	UP	18/30	1.606±0.334	=	18/30	2.217±0.501	UP	17/30
**miR-21**	10.180±3.602	UP	27/30	9.694±2.343	UP	27/30	13.614±5.270	UP	28/30
**miR-26a**	1.080±1.346	=	21/30	−5.974±0.143	DOWN	28/30	−1.450±0.964	=	19/30
**miR-27a**	1.419±0.339	=	22/30	2.995±0.489	UP	23/30	2.923±0.764	UP	23/30
**miR-31**	−3.142±0.775	DOWN	19/30	−8.838±1.471	DOWN	25/30	−1.891±1.041	=	11/30
**miR-34a**	1.029±0.674	=	15/30	2.205±0.983	UP	15/30	1.928±1.481	=	14/30
**miR-101**	−1.116±0.675	=	20/30	−2.466±0.209	DOWN	18/30	−2.656±0.241	DOWN	19/30
**miR-137**	−3.681±0.075	DOWN	24/30	−6.175±0.031	DOWN	29/30	−10.929±0.308	DOWN	29/30
**miR-182**	−1.049±1.394	=	12/30	1.924±1.923	=	11/30	4.737±1.756	UP	21/30
**miR-221**	−1.431±0.951	=	15/30	−1.267±0.733	=	14/30	−2.532±0.447	DOWN	18/30
**miR-222**	−10.230±0.194	DOWN	26/30	−1.982±0.674	=	10/30	−12.987±0.152	DOWN	28/30
**miR-330**	−4.765±0.228	DOWN	24/30	−1.715±0.432	=	14/30	−5.882±0.156	DOWN	24/30
**miR-519d**	−4.813±0.238	DOWN	22/30	−3.552±0.220	DOWN	20/30	−2.421±0.326	DOWN	17/30

a Status is determined according to Median Fold Change; ^b^ Number of GBMs showing the modulation out of a total of 5. FC: Fold change; UP: up-regulated (FC ≥2.0); DOWN: down-regulated (FC <−2.0);  = : not deregulated.

The remnant miRNAs (miR-7, miR-9, miR-9*, miR-21, miR-137, miR-519d) showed the same expression profile in the three groups even if differences in the level of up- or down-regulation could be observed (Table 2).

## Discussion

GBM is the most aggressive brain tumor that may occur in adults. Nevertheless, there were improvements in surgery, radiotherapy, chemotherapy, and “target therapy” [15], while its prognosis remains poor [13], [16]. MicroRNAs expression seems to play an important role in cancer development and progression and could be a possible target for molecular therapy [17]. For this reason, identifying a miRNAs profile in GBM could be very useful in developing new drugs and therapeutic approaches. The starting material and samples used as reference control are two crucial points for expression study design. In a previous study, the authors demonstrated that miRNAs analysis in GBM is feasible in FFPE samples, as well as in fresh/frozen ones [5]. Due to the difficulty of gathering non-neoplastic brain specimens, in literature, there are different samples chosen as reference control in miRNAs expression analysis (e.g. normal adjacent tissues [6]–[8] or epileptic samples [11], [17]). Moreover, several commercial pools of RNAs obtained from normal brain tissues were available, such as the Ambion FirstChoice® Human Brain Reference RNA [6], [16]. This situation led each group to arbitrarily choose a reference, sometimes obtaining different miRNAs expression profiles according to selected control [18].

In our study, we investigated if miRNAs expression profiles obtained using different non-neoplastic controls are comparable or not. For this reason, normal samples adjacent the tumor, commercial reference (FirstChoice® Human Brain Reference RNA – Ambion), and epileptic samples were used. Although microarrays are a widescreen and powerful method for miRNAs analysis, we focused on the 19 miRNAs previously analyzed and validated in order to determine the feasibility of analysis starting from both fresh frozen and FFPE tissues [5].

Before analyzing miRNAs expression data, some technical issues regarding the present study should be considered. The mean age of the epileptic group was significantly different from that of the others, as expected considering mean age of epilepsy onset. The commercial reference was a pool of RNAs obtained from multiple donors and several brain regions, while RNAs from other non-neoplastic groups (normal adjacent tissue and epileptic specimens) were not pooled together; for this reason, the replicates obtained from commercial reference represented technical replicates, while those obtained from the other groups were evaluated as biological replicates. Bearing in mind these issues, it should be considered that the aim of the present study was to determine whether GBM miRNAs profile shows differences using several non-neoplastic references. For this reason we reproduced three experimental conditions with normal adjacent tissues, commercial references or epileptic specimens as non-neoplastic controls.

The comparison between expression values of miRNA obtained in each of the three groups revealed good correlation values (>0.65). However, the correlation value was higher when comparing epileptic and commercial reference (R: 0.848). Meanwhile, when epileptic group and commercial reference were compared with normal adjacent the tumor, the correlation values were lower (R: 0.702 and 0.724, respectively). This could be due to the fact that the miRNAs expression profile of normal adjacent the tumor tissue could be influenced by the surrounding neoplastic cells, just as what happened during mRNA expression analysis experiments [19].

While comparing the median expression values of each miRNAs obtained in the three different groups, we observed some statistical significant differences (p<0.05) in several miRNAs (miR-7, miR-9, miR-10a, miR-10b, miR-26a, miR-27a, miR-31, miR-101, miR-137, miR-182, miR-519d).

Bearing in mind this evidence, we analyzed 19 miRNAs in a group of GBMs (thirty samples within the PERNO project cohort) using the three previously described references as non-neoplastic controls. We observed that miRNAs profiles obtained in these 30 GBMs were different according to the chosen control group. In fact, no differences were observed in 6 miRNAs, while 13 out of 19 (miR-10a, miR-10b, miR-17, miR-20a, miR-26a, miR-27a, miR-31, miR-34a, miR-101, miR-182, miR-221, miR-222, and miR-330) showed a different modulation in GBM depending on a selected reference, considering a cutoff of 2-fold change. Moreover, it should be noticed that, even in those miRNAs showing a comparable status in the three groups, differences in fold change values can be observed (e.g. miR-7, miR-137).

The differences in expression of some miRNAs in comparison with other studies could be due to: 1) the enrichment in neoplastic cells could give discrepant results with those obtained without performing dissection [5]; 2) different reference controls could lead to different miRNAs profiles as demonstrated in this study. An example was the study by Malzkorn et al. [10] in which miR-9, miR-17, miR-20a, and miR-21 showed an increased expression in recurrent GBMs compared with primary grade II tumors. Although a splendid approach and technique were used in the study, it is not advisable to compare these results with ours, both in agreement (e.g. mir-20a) and not (e.g. miR-9), because of a different reference (primary grade II tumors) used by Malzkorn et al. for determining the modulation of selected miRNAs.

Even though only 19 miRNAs were here considered, it is reasonable to hypothesize that the same discrepancies could be observed analyzing any miRNAs.

In conclusion, the present study shows that comparing miRNAs profiles obtained using different non-neoplastic controls is not recommended for several reasons: 1) the physiological differences in mean age that could be observed between different groups (e.g. epileptic specimens have a mean age lower than normal adjacent the tumor samples); 2) technical issues: e.g. a commercial reference is usually obtained pooling together several non-neoplastic RNAs (technical variability), while RNAs obtained from normal adjacent the tumour or epileptic specimens are not usually pooled together (biological variability); 3) different selected non-neoplastic groups could have real different miRNAs expression values. Having considered that the number of GBMs analyzed in this study was too small for determining a conclusive miRNAs profile (study in progress), we emphasized that the results of miRNAs profile in GBMs are strictly dependent on the non-neoplastic reference.

## Supporting Information

Figure S1 miRNAs profile in 30 GBMs compared with the three different non-neoplastic references. Lines in correspondence of Median FC  = +2 and −2 indicate the cut off for up- or down-regulation, respectively. Bars indicate FC median errors. FC, Fold change; N-Ad, Normal adjacent the tumor; Ref, Commercial reference; EP, epileptic.
(TIF)

Table S1 Median expression values obtained in the three different groups. *p-values were obtained using Kruskal-Wallis test.
(DOC)
