# Supplementary figures and images for: Definition of miRNAs Expression Profile in Glioblastoma Samples: The Relevance of Non-Neoplastic Brain Reference
Source: PLoS One. 2013 Jan 29;8(1):e55314. doi: 10.1371/journal.pone.0055314 (PMC3558478; doi:10.1371/journal.pone.0055314)

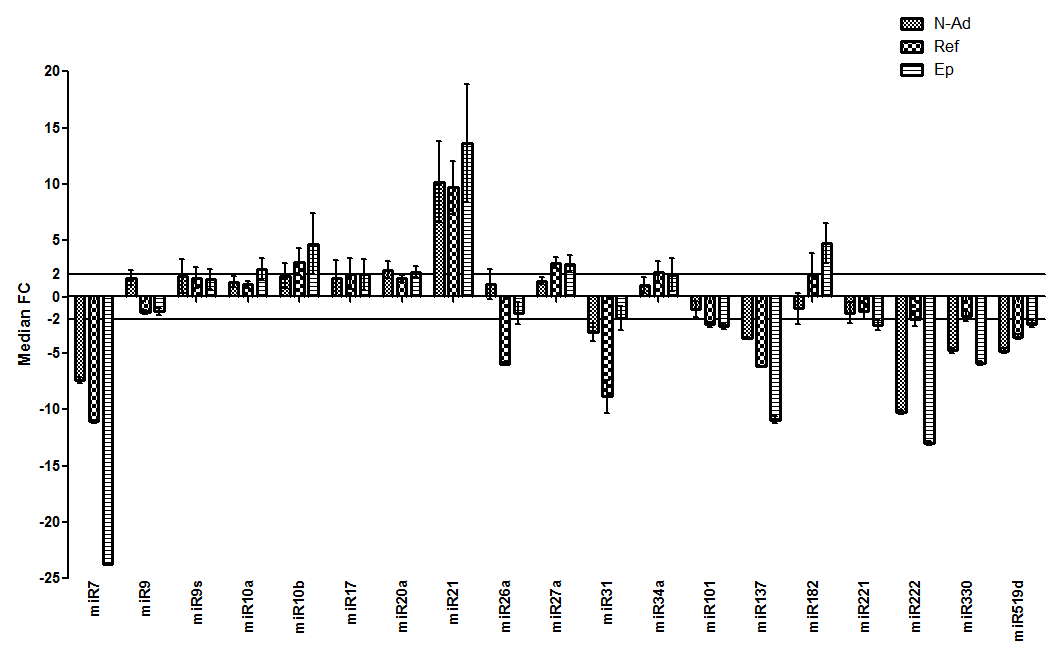

Supplement: Figure S1 — miRNAs profile in 30 GBMs compared with the three different non-neoplastic references. Lines in correspondence of Median FC = +2 and −2 indicate the cut off for up- or down-regulation, respectively. Bars indicate FC median errors. FC, Fold change; N-Ad, Normal adjacent the tumor; Ref, Commercial reference; EP, epileptic. (TIF) [file pone.0055314.s001.tif]
