# Supplementary material for: Definition of miRNAs Expression Profile in Glioblastoma Samples: The Relevance of Non-Neoplastic Brain Reference
Source: PLoS One. 2013 Jan 29;8(1):e55314. doi: 10.1371/journal.pone.0055314 (PMC3558478; doi:10.1371/journal.pone.0055314)
Supplement: Table S1 — Median expression values obtained in the three different groups. *p-values were obtained using Kruskal-Wallis test. (DOC) [file pone.0055314.s002.doc]

| **miRNAs** | **Median Expression Values** | | | **p-value*** |
| --- | --- | --- | --- | --- |
|  | *Normal Adjacent Tumor ± Median Error* | *Ambion Brain Reference ± Median Error* | *Epileptic Tissue ± Median Error* |  |
| **miR-7** | 1.441 ± 0.336 | 2.255 ± 1.515 | 4.517 ± 0.660 | p<0.01 |
| **miR-9** | 15.898± 3.508 | 45.191 ± 9.489 | 24.512 ± 3.744 | p<0.05 |
| **miR-9*** | 1.388± 0.876 | 4.767 ± 1.504 | 2.118 ± 0.572 | NS |
| **miR-10a** | 0.422± 0.249 | 0.706 ± 0.332 | 0.207 ± 0.116 | p<0.05 |
| **miR-10b** | 0.356± 0.203 | 0.258 ± 0.193 | 0.086 ± 0.118 | p<0.05 |
| **miR-17** | 0.031± 0.038 | 0.059 ± 0.003 | 0.051 ± 0.015 | NS |
| **miR-20a** | 0.039± 0.024 | 0.094 ± 0.001 | 0.049 ± 0.020 | NS |
| **miR-21** | 0.910± 0.814 | 2.473 ± 0.313 | 0.974 ± 0.668 | NS |
| **miR-26a** | 2.699± 0.698 | 17.851 ± 1.429 | 4.649 ± 0.779 | p<0.01 |
| **miR-27a** | 0.541± 0.166 | 0.402 ± 0.036 | 0.201 ± 0.075 | p<0.05 |
| **miR-31** | 0.077± 0.072 | 0.366 ± 0.091 | 0.062 ± 0.017 | p<0.05 |
| **miR-34a** | 0.833± 0.512 | 0.933 ± 0.355 | 0.759 ± 0.212 | NS |
| **miR-101** | 0.254± 0.085 | 0.632 ± 0.053 | 0.444 ± 0.171 | NS |
| **miR-137** | 0.316± 0.099 | 0.663 ± 0.226 | 1.042 ± 0.298 | p<0.01 |
| **miR-182** | 0.232± 0.131 | 0.263 ± 0.091 | 0.093 ± 0.029 | p<0.01 |
| **miR-221** | 3.753± 0.701 | 2.914 ± 0.999 | 5.018 ± 0.907 | NS |
| **miR-222** | 19.427± 9.525 | 6.466 ± 2.036 | 10.655 ± 6.789 | NS |
| **miR-330** | 0.691± 0.217 | 0.309 ± 0.053 | 0.371 ± 0.290 | NS |
| **miR-519d** | 0.644± 0.259 | 0.742 ± 0.248 | 0.309 ± 0.119 | NS |
